# A Novel Recovery Method of Soft X-ray Spectrum Unfolding Based on Compressive Sensing

**DOI:** 10.3390/s18113725

**Published:** 2018-11-01

**Authors:** Nan Xia, Yunbao Huang, Haiyan Li, Pu Li, Kefeng Wang, Feng Wang

**Affiliations:** 1 Provincial Key Laboratory of Computer Integrated Manufacturing, Guangdong University of Technology, Guangzhou 510006, China; xianan_tech@163.com (N.X.); huangyb@gdut.edu.cn (Y.H.); lipu_300@126.com (P.L.); gdut_wkf@163.com (K.W.); 2 School of Physics and Electrical Engineering, Shaoguan University, Shaoguan 512005, China; 3 Laser Fusion Research Center, China Academy of Engineering Physics, Mianyang 621900, China; Wangfeng7566@163.com

**Keywords:** spectrum unfolding, compressive sensing, sparse representation, lasso regression, soft X-ray spectrometer, spectral measurement

## Abstract

In the experiment of inertial confinement fusion, soft X-ray spectrum unfolding can provide important information to optimize the design of the laser and target. As the laser beams increase, there are limited locations for installing detection channels to obtain measurements, and the soft X-ray spectrum can be difficult to recover. In this paper, a novel recovery method of soft X-ray spectrum unfolding based on compressive sensing is proposed, in which (1) the spectrum recovery is formulated as a problem of accurate signal recovery from very few measurements (i.e., compressive sensing), and (2) the proper basis atoms are selected adaptively over a Legendre orthogonal basis dictionary with a large size and Lasso regression in the sense of ℓ1 norm, which enables the spectrum to be accurately recovered with little measured data from the limited detection channels. Finally, the presented approach is validated with experimental data. The results show that it can still achieve comparable accuracy from only 8 spectrometer detection channels as it has previously done from 14 detection channels. This means that the presented approach is capable of recovering spectrum from the data of limited detection channels, and it can be used to save more space for other detectors.

## 1. Introduction

Investigation of laser-driven inertial confinement fusion (ICF) is of great significance in the search for the ideal energy resource, as well as the application of fundamental scientific research [1]. ICF is a process in which nuclear fusion reactions are initiated by heating and compressing a fuel capsule containing a mixture of Deuterium and Tritium [2]. The purpose of laser fusion diagnostics is to reveal the state and behavior of the target plasma by measuring the characteristics of the plasma radiation and fusion reaction products, and then give insight into the absorption mechanism and regular characteristics of the laser energy.

**Figure 1:**
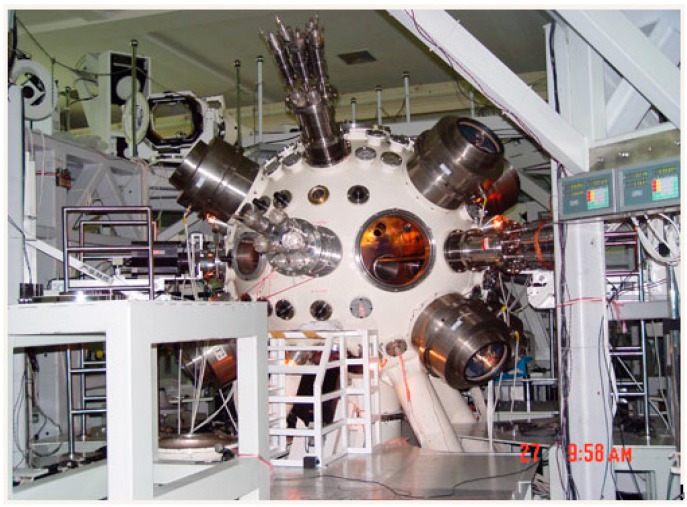
The ICF experimental facility.

Figure 1 is the ICF experimental equipment Shenguang II. In the ICF experiment, the laser plasma emits strong X-ray radiation whose energy spectrum is mainly concentrated in the soft X-ray energy region, and its photon energy ranges from tens of electron volts to thousands of electron volts. The spectrometer will detect the power spectrum and then transmit the detected signals to the computer, and the spectrum information of the target can be obtained [3]. The spectral information can be used to research the implosion process with the plasma. Thus, it is crucial to investigate the soft X-ray radiation spectrum in the ICF diagnosed experiment.

The solution of the unfolding problem is an ever-present issue in X-ray spectrometry. To recover the original spectrum it is necessary to use the detector response function, by solving the so called inverse problem [4]. There are many different methods that can be used to solve this problem, and each different approach leads to a different unfolding method and a different approximate solution [5], but in general the strategy is familiar: Search for a solution that is close to a reasonable estimate of the spectrum which could give a good data fit, without over-fitting or under-fitting.

The maximum entropy unfolding technique [4] solves the inverse problem by imposing a set of physical constraints artificially. The stochastic methods [5], such as the Monte Carlo methods [6], Genetic algorithms [5], and Neural networks [7], are used to derive the solution spectrum, and are successful in some specific applications. The weighted method which modifies the portion of each spectrometer response was introduced by Fehl [8] to recover the radiation flux. However, the combined flat response is not perfectly smooth, and errors will be introduced into the recovered radiation flux. The basis function method uses the linear combination of the basis function [9] to express the spectrum. There are mainly two techniques to this method.

One technique is selecting the reasonable basis function to reconstruct the spectrum. The response function [10], Piece-wise B-spline [10,11], and Gaussian Bumps, among others [12,13,14], are shapes of the basis function frequently used in spectrum unfolding experiments. By using these basis functions, basis atoms should be selected in some certain rules in advance. Thus, these selected basis atoms might not be enough and fixed, and these basis functions may have difficulty presenting the spectrum signal.

The other technique is choosing an appropriate calculation method to determine coefficients of the basis function. The following calculation methods are commonly used at present: Iteration based on maximum entropy [11,15,16], the Bayesian theorem [16], singular value decomposition (SVD) [10], and Least Squares (LS) [10], among others. Since these coefficients are the solution in the sense of the ℓ2 norm, which are calculated over the fixed basis dictionary, the solution may be under-fitting with some measured data, and these methods cannot recover the spectrum precisely.

These methods may need more measurement data to achieve good recovery performance for a spectrum. Nevertheless, with the laser beams increasing, it is too difficult to obtain more detection channel data with the soft X-ray spectrometer, which yields some difficulties in a practical ICF facility. Since there is a lot of other physical information that needs measured to research the soft X-ray, including the temperature and radiation, the possible install positons and the opening of the center target for spectrometers is limited, and their orientation also leads them to interfere with each other. It is hard to add detection channels to achieve good performance in recovering the spectrum. Thus, it is necessary to find a new recovery method to unfold a spectrum with finite detection channels.

Compressive sensing (CS), proposed by Donoho and Candès [17], is a new method to reconstruct signals from significantly fewer sampling points than is required by traditional methods. In recent years, compressive sensing has attracted considerable attention in areas of medical imaging (MI) [18], Analog-digital Conversion [19], Computational Biology [20], Computer Graphics [21], and other aspects. Owing to the significance of compressive sensing, CS can be applied in this paper to solve the problem of spectrum unfolding with limited detection channels data, and achieve the following:

(1) The spectrum recovery is formulated as a problem of an accurate signal recovering from a few measurements (i.e., compressive sensing), which applies the measurement matrix to convert the spectrum signal into a voltage signal, and enables the signal be reconstructed with a small amount of measured data.
(2) The proper basis atoms are selected adaptively over the Legendre orthogonal basis dictionary with a large size and Lasso regression in the sense of the ℓ1 norm, which enables the spectrum to be recovered with high accuracy from the small amount of measured data of the limited detection channels.
(3) By employing this method, since the soft X-ray spectrometer may recover the spectrum with limited detection channel data, it provides the possibility of saving space for other detectors.

The rest of this paper is organized as follows. In the next section, we introduce the soft X-ray spectrometer and the previous principle of the energy spectrum recovery with multi-channels, followed by a brief review of compressive sensing theory. Section 3 gives the entire formulation of this novel method of spectrum unfolding based on compressive sensing. Then in Section 4 we discuss the numerical experiments of spectrum recovery, and the results which support our viewpoints. Finally, the paper comes to an end with a summary of some significant conclusions.

## 2. Soft X-ray Spectrometer and Compressive Sensing

In this section, we first present the soft X-ray spectrometer in the ICF experiment, including its structure, operating principle, and drawbacks in the process of spectrum unfolding. Then we demonstrate compressive sensing theory through discussion of sparse representation, the measurement matrix, and sparse coefficients reconstruction.

### 2.1. Soft X-ray Spectrometer

In indirect-drive inertial confinement fusion (ICF) experiments, high power laser beams irradiate the high-Z inner wall of the hohlraum and the energy is converted into soft X-ray radiation, which is used to drive the capsule located inside the hohlraum to implode, or to irradiate a package of materials to study their properties [9,22,23]. In order to investigate the physical process of ICF, it is important to measure the radiation spectrum distribution of the soft X-ray. Multi-channel spectrometers composed of filtered X-ray diodes (XRDs) are routinely used in ICF experiments to measure the radiation flux and recover the spectrum from the laser produced soft X-ray radiation source [9,24]. This kind of spectrometer includes the Dante on Omega and NIF (National Ignition Facility) in the U.S (Rochester, NY, USA). Ref. [13,24,25], the DMX in France [26], and the soft X-ray spectrometer used on Shenguang laser facilities in Mianyang, China [27].

**Figure 2:**
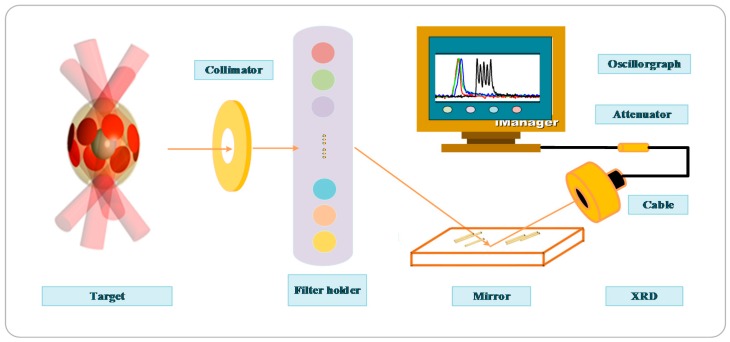
Soft X-ray spectrometer layout.

The soft X-ray spectrometer works using the filter method shown in Figure 2, and it’s structure contains the following parts: Collimator, filter, mirror, XRD, cable, attenuator, and oscillograph [10]. The filtering method is composed of a windowless soft X-ray diode as a detector, and it can be regarded as a soft X-ray detection system. Firstly, the energy spectrum from the soft X-ray is divided into several energy channels with different combinations of filters and mirrors [27], in which the thickness of the filters can influence the soft X-ray transparency, and the mirrors have characteristics that can cut off the high energy tails. Then, the detecting response may be obtained to measure the spectral signal. Depending on these detecting responses, the XRD of the high voltage power supplying system can transmit the spectral signal to the digital oscilloscope through the microwave cable, and the data can be collected. Finally, voltage signals may be displayed on a high-speed oscilloscope.

**Figure 3:**
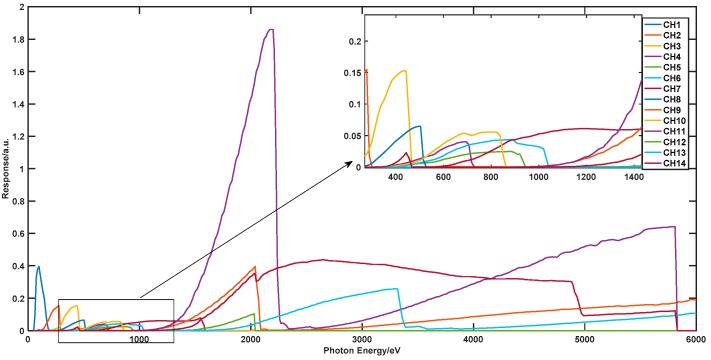
Fourteen channel responses of the soft X-ray spectrometer.

Taking a 14 channel spectrometer as an example, the detecting response is shown in Figure 3.

In Figure 3, normalized channel response functions of a 14-channel spectrometer are depicted. This spectrometer covers photon energy from 50 eV to 6000 eV. Different channels from the filtered X-ray diode array use filters and mirrors made of different materials to realize roughly band-pass measurements [9]. The signal of the channels from the filtered XRD arrays is determined by the following integral:

(1) $$D_{k}=\int M_{k}\left(E\right)S\left(E\right)dE,\;k=1,\ldots ,m$$

where m is the number of detecting channels, E is the photon energy, S(E) is the soft X-ray spectrum, $M_{k}\left(E\right)$ is the response function of the kth channel, and $D_{k}$ is the voltage signal from the kth channel recorded by a high speed oscilloscope. Equation (1) can usually be deemed as the first kind Fredholm integral equation, and spectrum recovery can be obtained by solving this inverse problem.

The recovery method [10] based on a basis function was introduced to approach the original spectrum with the linear combination of basis function ${\left\{B_{j}\left(E\right)\right\}}_{j=1,2,\ldots ,N}$, such as the Piece-wise B-spline function. ${\left\{S_{j}\right\}}_{j=1,2,\ldots ,N}$ are coefficients, in which N is the number of basis atoms, and the errors are generated. It can be described as

(2) $$S\left(E\right)=\sum_{j=1}^{N}S_{j}B_{j}\left(E\right)+\varepsilon$$

The purpose of the diagnosis is to recover the radiation spectrum S(E) from the known response function $M_{k}\left(E\right)$ and voltage signal $D_{k}$. Combine Equations (1) and (2) and the voltage signal $D_{k}$ is written as

(3) $$D_{k}=\overset{N}{\underset{j=1}{\sum }}S_{j}\int M_{k}\left(E\right)B_{j}\left(E\right)dE+\varepsilon$$

Let $R=\int M_{k}\left(E\right)B_{j}\left(E\right)dE$, then the voltage signal $D_{k}$ is expressed as

(4) $$D_{k}=\overset{N}{\underset{j=1}{\sum }}S_{j}R$$

The coefficient vector $S_{j}$ can be calculated based on the Least Square algorithms. The spectrum S(E) can be determined through Equation (2). In this method, the basis atom is chosen factitiously in the fixed basis function which may have the limited basis atom, to recover spectrum. More measurement data would be needed to calculate the coefficient vector, otherwise there may be under-fitting in the process of spectrum recovery. However, the number of detection channels is enslaved to the spectrometer structure. There is much physical information that needs measured to research the soft X-ray, including the temperature and radiation, and the install positon and the opening of the center target leaving for the detecting channels is limited, with their orientations also interfering with each other. This makes it hard to add detection channels for more measurement data.

To clearly comprehend this, we supposed that according to the response data from 14 detection channels, which is shown in Figure 3, the basis atoms factitiously selected are fixed and the number of the basis atoms is 14. With this method, these coefficients can be calculated over a fixed basis dictionary and solutions are in the sense of the ℓ2 norm, which may not be computed accurately with limited measured data. The recovery spectrum can be easily under-fitting.

Owing to the significance of compressive sensing, a method to reconstruct signals from significantly fewer sampling points than traditional methods is required. We present a novel spectrum unfolding method based on compressive sensing, which may enable basis atoms to be selected adaptively over a large size of basis dictionary with high-order basis atoms, and the spectrometer may achieve high accuracy spectral information from limited detection channels.

### 2.2. Compressive Sensing Method

Compressive Sensing, also known as compressive sampling, is a novel sensing paradigm that is widely used in data acquisition. CS theory asserts that one can recover certain signals and images from far fewer samples or measurements than traditional methods use [28]. To be more specific, the idea of compressive sensing is to reconstruct a signal from a few samples by exploring it’s sparse characteristics over some kinds of representation basis, such as the Fourier representation basis, Discrete cosine basis, and Discrete wavelet basis, among others. Compressive sensing generally consists of the following parts: Sparse representation, a measurement matrix, and sparse coefficients reconstruction.

#### 2.2.1. Sparse Representation

The theory of sparse representation was first proposed in signal field and later widely applied to solve undetermined equations [29]. It reveals an interesting phenomenon, that a function or signal can be represented with just a few significant terms from a set of base functions. This means there are only a few large (in magnitude) coefficients corresponding to the base functions, and most of them are zero or they may be ignored with minimal perceptual loss.

Given the set of sampling points $x={\left[x_{1},\;x_{2},\ldots ,x_{n}\right]}^{T}$ on the interval [a,b], and the corresponding actual signal $F={\left[F(x_{1}),\;F(x_{2}),\ldots ,F(x_{n})\right]}^{T}$, any signal $F\in {\mathbb{R}}^{n\times 1}$ can be represented by a linear combination of a complete basis:

(5) $$F=\overset{N}{\underset{i=0}{\sum }}\theta_{i}\varphi_{i}\left(x\right)=\Phi \cdot \Theta$$

where the sparse basis matrix (so-called dictionary) $\Phi \in {\mathbb{R}}^{n\times n}$ is composed of a set of basis atoms, and $\Theta \in {\mathbb{R}}^{n\times 1}$ is the coefficient vector. If Θ has only ρ≪n non-zero elements, in which ρ sparseness represents the number of non-zero elements, Θ is named as the sparse coefficient vectors. Additionally, if ρ is small enough, the signal F may be accurately reconstructed by a few sampling points.

#### 2.2.2. Measurement Matrix

Supposing a measurement matrix $A\in {\mathbb{R}}^{m\times n}\left(m\ll n\right)$, the function is reduced from the n-dimension to m-dimension by linear transformation as follows:

(6) Y=A·F=A·Φ·Θ=Ψ·Θ

where $\Psi \in {\mathbb{R}}^{m\times n}$ is obtained by A timing Φ, and can be called a sensing matrix. $Y\in {\mathbb{R}}^{m\times 1}$ is the transformed vector. This theory asserts that the function $F\in {\mathbb{R}}^{n\times 1}$ can be recovered by. $Y\in {\mathbb{R}}^{m\times 1}$ [30] under two conditions. The first one is the sparsity, which demands that the function should be sparsely represented on the dictionary Φ. The other is the incoherence, which means these two matrices need to satisfy the condition of the Restricted Isometry Property (RIP); that is, the measurement matrix A should be incoherent with dictionary Φ, and it can be described as

(7) $$\left(1-\delta_{s}\right)\|\Theta \|{}_{2}^{2}\leq \|\Psi \Theta \|{}_{2}^{2}\leq \left(1+\delta_{s}\right)\|\Theta \|{}_{2}^{2}$$

The restricted isometry constant $\delta_{s}(0<\delta_{s}<1)$ is defined as the smallest constant for which this property holds for all sparse coefficient vectors Θ. It is difficult to set up a matrix that satisfies the RIP condition, or verify the matrix satisfies the RIP condition. The non-coherent property was proposed by Candès and Wakin [17]; that is, if the measurement matrix A is linearly independent of the dictionary Φ, or the correlation of both is weak, the two matrices will satisfy the RIP criterion with high probability. It has been proven that random matrices are largely incoherent with any fixed basis [17].

Since the dictionary Φ is reasonably designed, only the measurement matrix $A\in {\mathbb{R}}^{m\times n}$ may be varied. There are several measurement matrices to construct the measurement matrix; examples of these matrices include Circulant [31], Toeplitz [32], Chirp sensing [33], Gaussian, and Uniform random matrices. As the random matrix is easy to implement in the current spectral recovery experiment and can be incoherent with a fixed basis, it was used to construct the measurement matrix. With this matrix, the measurement matrix can be selected randomly, and random matrices A may be largely incoherent with any fixed basis Φ.

#### 2.2.3. Sparse Coefficients Reconstruction

In order to recover the function with limited sampling points, we must seek the sparse representation over the dictionary. The sparse coefficient Θ can be obtained by solving the inverse problem of Equation (6) with an appropriate compressive sensing reconstruction algorithm, which can be seen as solving the following problem:

(8) $$\left(p_{0}\right)\;:\min{\left\|\Theta \right\|}_{0}.\;\;s.t.\;\|Y-\Psi \Theta \|{}_{2}^{2}\leq \varepsilon$$

The above problem is called the $p_{0}$ question, which is known as an NP (Non-Deterministic Polynomial) hard problem. To solve this problem, the Greedy algorithm, Bayesian category [34], and Convex Relaxation Technique have been proposed. The Greedy algorithm is efficient, but has poor performance in anti-noise; examples include the Matching Pursuit (MP) [35] and Orthogonal Matching Pursuit (OMP) [36], among others. The Bayesian category [37] can balance between small recovery error and short recovery time in the large scale problem, with an example being the Bayesian Relevance Vector Machine (BCS-RVM) [38]. Convex Relaxation Techniques, such as Basis Pursuit (BP) [39] and Lasso regression [40], have an excellent ability in basis atom selection and coefficient shrinkage. The latter algorithm has a higher accuracy and its performance can be maintained even with added noise in the system. In this paper, we prefer to apply lasso regression to obtain the coefficient vector Θ, which cannot only compress most of the coefficients to zero, but also avoid over-fitting.

To make this possible, Lasso regression relaxes the ℓ0 norm to the ℓ1 norm.

(9) $$\left(p_{1}\right):\min{\left\|\Theta \right\|}_{1}.\;\;s.t.\;{\left\|Y-\Psi \Theta \right\|}_{2}^{2}\leq \varepsilon$$

The ℓ1 norm question is a convex problem which has the most similarity to the ℓ0 norm. It can be written as Lagrange multiplier:

(10) $$\hat{\Theta }\left(\beta \right)=arg\min_{\Theta }\;{\left\|Y-\Psi \Theta \right\|}_{2}^{2}+\beta {\left\|\Theta \right\|}_{1}$$

The last equation is the famous Lasso (least absolute shrinkage and selection operator) model, which consists of a least square estimate and a ℓ1 norm penalty.

Where β≥0 denotes the penalty factor, it may be deemed as the weight between cost function ${\left\|Y-\Psi \Theta \right\|}_{2}^{2}$ and the penalty $\beta {\left\|\Theta \right\|}_{1}$. The larger the value β is, the more elements in $\beta {\left\|\Theta \right\|}_{1}$ will be close to zero. This means that $\beta {\left\|\Theta \right\|}_{1}={\left\|\beta \Theta \right\|}_{1}$ can contribute to the sparse solution in terms of ℓ1 norm regularization. The accuracy of reconstruction also depends on the appropriate value of β. Thus, it is necessary to find the “coefficient path” which represents the relationship between the solution $\hat{\Theta }\left(\beta \right)$ and β.

There are a lot of operators for solving the Lasso problem, such as quadratic programming [40] and the least angle regression lasso (LAR-Lasso) [41] algorithm. Quadratic programming uses the ℓ1 norm penalty on the regression coefficients, and it tends to produce simpler models [42]. LAR-Lasso is a method that can be viewed as a vector-based version of lasso to accelerate the computations [42]. Zou et al. [43] proposed an efficient algorithm called LARS-EN to solve Lasso with a regularization parameter which is based on LARS. The algorithm complexity is equivalent to the complexity of least squares. It is particularly useful when the number of predictors is bigger than the observer number. We chose LARS-EN in this article and downloaded the Matlab toolbox from http://www2.imm.dtu.dk/projects/spasm/.

## 3. Spectrum Unfolding based on Compressive Sensing

**Figure 4:**
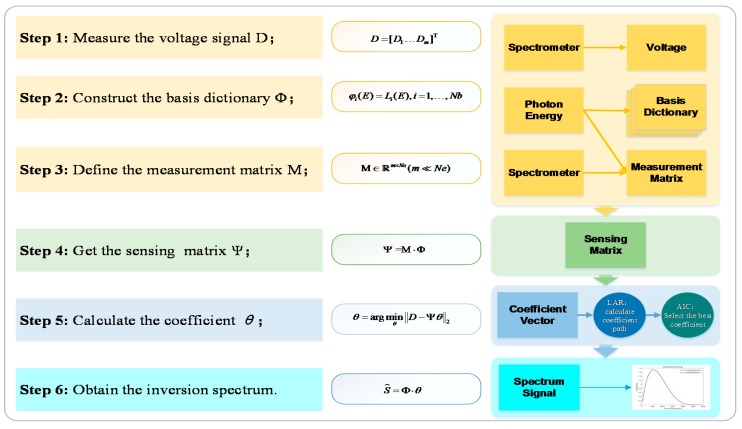
The process of spectrum unfolding based on compressive sensing.

In this section, spectrum recovery is formulated as a problem of accurate signal recovery from a few measurements; that is, compressive sensing. We will detail that the proper basis atoms are selected adaptively over a Legendre orthogonal basis dictionary with a large size and Lasso regression in the sense of the ℓ1 norm, which enables the spectrum to be recovered with high accuracy from the minimal measured data of the limited detection channels. The entire process of the spectrum unfolding method based on compressive sensing is given in Figure 4.

### 3.1. Sparse Representation of Spectrum with Legendre Polynomial

The spectrum signal $\hat{S}$ is represented by a sum of base functions with their coefficients, and it can be written as

(11) $$\hat{S}=\overset{N_{b}}{\underset{i=1}{\sum }}\theta_{i}\varphi_{i}\left(E\right)$$

and it’s matrix form is

(12) $$\hat{S}=\Phi \cdot \Theta$$

where $E={\left[E_{1},\;E_{2},\ldots ,E_{N_{e}}\right]}^{T}$ is the discrete equidistant point represented by the photon energy, and $N_{e}$ is the number of the photon energy point. ${\left\{\theta_{i}\right\}}_{i=1,2,\ldots ,N_{b}}$ are coefficients, $\Theta ={\left[\theta_{1},\;\theta_{2},\ldots ,\theta_{N_{b}}\right]}^{T}$ is the coefficient vector, ${\left\{\varphi_{i}\left(E\right)\right\}}_{i=1,2,\ldots ,N_{b}}$ are basis functions, $\Phi =\left[\varphi_{1},\;\varphi_{2},\ldots ,\varphi_{N_{b}}\right]$ is the set of base functions, and $N_{b}$ is the number of the basis function which determines the dictionary size. For $\varphi_{i}\left(E\right)$ is a polynomial, a set of orthogonality polynomials is commonly used as atoms to describe the signals in the theory of compressive sensing.

In the process of sparse representation of the spectrum, we prefer to choose the basis function which has orthogonal properties, like Legendre polynomials. The Legendre orthogonal basis function is good at expressing the spectral curve with high-order basis atoms, and it is easily constructed and calculated. By measuring with the sparse representation of the signal on the orthogonal basis, a few coefficients can be used to present spectrum unfolding. In addition, the Legendre polynomial is orthogonal with respect to the ℓ2 norm, which means in the interval [−1, 1], the energy range should be transferred into [−1, 1] in advance.

The Legendre polynomials can be determined by the recursive definition. Suppose that $L_{0}\left(E\right)=1,L_{1}\left(E\right)=E$, then

(13) $$\left(n+1\right)L_{n+1}\left(E\right)=\left(2n+1\right)L_{n}\left(E\right)-nL_{n-1}\left(E\right),\;\;n=1,2,\cdots ,N_{b}$$

The basis function of Legendre orthogonal polynomials can be defined as

(14) $$\varphi_{i}\left(E\right)=L_{i}\left(E\right),\;\;i=1,\ldots N_{b}$$

Given the sampling points of photon energy, the sparse basis matrix $\Phi \in {\mathbb{R}}^{N_{e}\times N_{b}}$ (so-called dictionary) composed by a set of atoms is written as follows:

(15) $$\Phi =\left[\begin{array}{ccc} L_{1}\left(E_{1}\right) & \cdots & L_{N_{b}}\left(E_{1}\right)\\ \vdots & \ddots & \vdots \\ L_{1}\left(E_{N_{e}}\right) & \cdots & L_{N_{b}}\left(E_{N_{e}}\right) \end{array}\right]$$

The basis dictionary can be generated with a large number of basis atoms. Due to high order expansion on the Legendre basis function and a plentiful basis dictionary, the proper basis atoms can be selected adaptively, and higher accuracy spectral information may be achieved.

### 3.2. Measurement Data for Sparse Spectrum Reconstruction

The measurement matrix $M\in {\mathbb{R}}^{m\times N_{e}}\left(m\ll N_{e}\right)$ is constructed by multiple sets of response values, which are generated by the corresponding detection channels. To obtain different response data, there are many configuration parameters that could be adjusted in each detection channel, such as the filter materials, the thickness of the filter, the installation of the plane mirror, and the size of the solid angle. The values for these configuration parameters depend on the experimental conditions, so they have to be chosen from the existing experimental devices. Examples of filter materials, of which there are almost ten more device varieties, such as Al, B, C, Ti, Cr, Fe, Ni, Cu, and Zn. As well as other configuration parameters, they can be selected in a definite range. The combination of all the configuration parameters can be made as a data set and labeled. p represents the number of all combinations. However, there are only m positions to install the detecting channels in the laser facility. There are many methods for taking m detection channels from p combinations. To improve the incoherence of the samplings, the random matrix is used to select m detection channels, and then the label is used to find out the configuration parameters. The spectrometer would be scaled by these configuration parameters to obtain the m response, and each response can be represented as a set of vectors which have $N_{e}$ samplings. Finally, the measurement matrix $M\in {\mathbb{R}}^{m\times N_{e}}$ can be constructed, and it can be represented as

(16) $$M=\left[\begin{array}{ccc} M_{1}\left(E_{1}\right) & \cdots & M_{1}\left(E_{N_{e}}\right)\\ \vdots & \ddots & \vdots \\ M_{m}\left(E_{1}\right) & \cdots & M_{m}\left(E_{N_{e}}\right) \end{array}\right]$$

After the measurement matrix M is constructed, it can be used to measure the original spectral signal and obtain the new converted signal. Based on the spectrum unfolding principle of the soft X-ray spectrometer and Equation (1) in the last section, it can be seen that the spectral signal can be converted into voltage signals $D={\left[D_{1},\;D_{2},\ldots ,D_{m}\right]}^{T}$. The conversion process can be described with Equation (17):

(17) $$\hat{D}=M\cdot \hat{S}=M\cdot \Phi \cdot \Theta =\Psi \cdot \Theta$$

where $\Psi \in {\mathbb{R}}^{m\times N_{b}}$ is the sensing matrix, which is a non-full rank matrix generated with measurement matrix $M\in {\mathbb{R}}^{m\times N_{e}}$ and basis dictionary $\Phi \in {\mathbb{R}}^{N_{e}\times N_{b}}$.

To make it clearer, we supposed that according to the response data from 14 detection channels, which are shown in Figure 3, the number of basis atoms $N_{b}$ can be set to 84 (this value depends on the compromise scheme based on the theoretical results in Reference [44]; i.e., let $N_{b}=6\;m$), which means the basis dictionary has a large number, and the highest order of the basis function is up to 83. This can be compared with the Piece-wise B-spline method referred to in the last section, and as it can see the spectrum, may be expressed more accurately in the large basis dictionary.

### 3.3 Sparse Coefficient Recovery for the Soft X-ray Spectrum

From Equation (17), the work recovering the spectrum is turned to solving the problem

(18) $$\Theta =arg\min_{\Theta }\;{\left\|D-\Psi \Theta \right\|}_{2}^{2},\;\;\;s.t.{\left\|\Theta \right\|}_{0}\leq \rho$$

Equation (18) is an NP hard problem, which has many groups of uncertain solutions. According to the brief of Section 2, Lasso regression can relax the ℓ0 norm to the ℓ1 norm, thus Equation (19) can be obtained:

(19) $$\Theta =arg\min_{\Theta }\;{\left\|D-\Psi \Theta \right\|}_{2}^{2},\;\;\;s.t.\overset{N_{b}}{\underset{i=1}{\sum }}{\left|\theta_{i}\right|}_{1}\leq t$$

Due to the sensing matrix $\Psi \in {\mathbb{R}}^{m\times N_{b}}$ containing a large number of high-order basis atoms, the spectrum may be represented precisely. It also has the trait of completeness and redundancy, which may make the spectrum-unfolding over-fitting. To solve the problem, Least Angle Regression (LAR) [45] is employed to calculate the entire path of solutions as t is varied, in which t should be adaptively chosen to minimize an estimate of expected prediction error. As the tuning parameter t varies, making t sufficiently small will cause some of the coefficients to be exactly zero, and sparseness ρ to be close to zero. Whereas, when the value t is increasing, the number of non-coefficients ρ is increasing, and the spectrum recovery may be more accurate. Next, Akaike’s Information Criterion (AIC) [46] is applied to trade-off the sparsity and the accuracy to select the best coefficient path, which can be defined as

(20) $$AIC={\left\|D-\Psi \Theta \right\|}_{2}^{2}+2r^{2}K\left(\Theta \right)$$

where K(Θ) presents the number of nonzero elements of Θ, and $r^{2}$ denotes the residual variance, which can be calculated by Equation (21):

(21) $$r^{2}=\frac{1}{\rho }\|D-\Psi \Psi^{+}D\|{}_{2}^{2}$$

where $\Psi^{+}$ is the Moore–Penrose pseudo-inverse of Ψ. $I_{best}$ is supposed to the index of the best vector. ${\left\{{\hat{\Theta }}^{\left(z\right)}\right\}}_{z=1,2,\ldots ,Z}$ is generated by LARS-EN, ${\hat{\Theta }}^{\left(z\right)}$ is the coefficient vector calculated in the zth iteration, and Z is the maximum number of iterations. Therefore, we can have

(22) $$AIC^{\left(I_{best}\right)}=\underset{1\leq z\leq Z}{min}\left\{AIC^{\left(z\right)}=\|D-\Psi {\hat{\Theta }}^{\left(z\right)}\|{}_{2}^{2}\right\}+2r^{2}K\left({\hat{\Theta }}^{\left(z\right)}\right)$$

Subsequently, the best coefficient vector ${\hat{\Theta }}^{\left(I_{best}\right)}$ can be acquired, and the best reconstruction voltage signal can be expressed as

(23) $${\hat{D}}_{best}=\Psi \cdot {\hat{\Theta }}^{\left(I_{best}\right)}$$

Finally, the best recovery spectrum ${\hat{S}}_{best}$ can be calculated as

(24) $${\hat{S}}_{best}=\Phi \cdot {\hat{\Theta }}^{\left(I_{best}\right)}$$

### 3.4. Overview of the Soft X-ray Spectrum Unfolding Process

The process of the proposed spectrum unfolding method based on compressive sensing is shown in Figure 4. The spectrum recovery is an inverse problem, which depends on the measured voltage signal D from the soft X-ray spectrometer to calculate the spectral information. At first, the voltage signal D is obtained by the oscilloscope, and then the basis dictionary, which can sparsely represent the spectrum, is built based on the Legendre polynomial; the range of the photon energy E should be mapped into [−1, 1] in advance. To improve the incoherence of the samplings, the measurement matrix M can be constructed by modifying the configuration parameters from the detection channels at random; these random parameters include the filter material, the thickness of the filter, the installation of the plane mirror, and the size of the solid angle. Next, the sensing matrix can be obtained by multiplying the measurement matrix M and Φ. Finally, LARS-EN can be employed to calculate the coefficient path, and the AIC criterion is adopted to select the best coefficient vector, and thus the spectrum will be recovered.

## 4. Numerical Experiments

In this section, we provide two groups of contrasting experiments to prove that this novel method enables accurate signal recovery from a few measurements.

### 4.1. Accuracy Assessment Criteria

To verify the spectrum unfolding method, we conducted self-inspection of numerical experiments. In order to imitate the real experiment, we input the known spectrum S, called the original spectral signal, then we acquired the voltage signal value by the simulation of the soft X-ray spectrometer. To solve the inverse question, the value of voltage signal could be deemed as the input vector, and the recovery spectrum $\hat{S}$ could be obtained by the spectrum unfolding method. Comparing the two sets of data S and $\hat{S}$, we could determine the spectral recovery accuracy based on this spectrum unfolding method. For this experiment, it was very important to verify the accuracy of the spectrum recovery.

In addition to the self-inspection of numerical experiments, we also reproduced the experiment of the recovery performance with different spectrum unfolding methods, in which both the Piece-wise B-spline and Gaussian Bump methods were used in past research. We contrasted these two methods with the compressive sensing method in recovery performance. Furthermore, in the experiment we also explored the accuracy of the three methods with a small amount of measured data from the limited detection channels of the soft X-ray spectrometer.

The recovery error is a metric to evaluate the error between the original spectrum signal and the recovered one. In order to calculate the recovery error, the following formulas were used in this literary work:

1. Root mean squared error (RMSE)

Since the actual spectrum of the benchmark functions was known, the actual errors at any points could be computed. The RMSE is given by

(25) $$RMSE=\sqrt{\frac{1}{n}\sum_{k=1}^{n}{\left(S^{\left(k\right)}-{\hat{S}}^{\left(k\right)}\right)}^{2}},\;k=1,2,\cdots ,n$$

where $S^{\left(k\right)}$ is the actual spectrum, ${\hat{S}}^{\left(k\right)}$ is the spectrum unfolding at the kth test point, and n is the number of test points.

2. Mean absolute error (MAE)

The mean absolute error is given by

(26) $$MAE=\frac{1}{n}\sum_{k=1}^{n}\left|S^{\left(k\right)}-{\hat{S}}^{\left(k\right)}\right|,\;k=1,2,\cdots ,n$$

### 4.2. Experimental Settings

To show the robustness of the method, two types spectrum were regarded as the original spectrum; that is, the Plank spectrum and Triple-peak spectrum, which are commonly used in spectrum recovery experiments. In order to see the recovery performance, three methods were applied to contrast these. The first one is proposed in this paper based on the compressive sensing method, the second is the Piece-wise B-spline spectral method, and the third is the Gaussian Bump. The basic parameter settings based on the compressive sensing method were as follows: The photon energy ranged from 50 eV to 6000 eV, the number of sampling points for photon energy was $N_{e}=500$, and the number of the Legendre basis was $N_{b}=6\;m$. To better illustrate the capacity of spectrum unfolding with limited detection channels, the number of detecting channels m was regarded as the experimental variable. We assumed that the number of detection channels in spectrometers is up to 14, and that the result of spectrum unfolding accuracy may change as the number decreases; hence, we set m=14, 12, 10, 8. The experiment was run on a desktop computer with Intel Quad-core CPU, a frequency of 4.2 GHz, and 8 G memory, and MATLAB R2016a was used to validate the efficiency of the proposed approach.

### 4.3. Experimental Results and Analysis

Experimental figures show the spectrum recovery performance with three different spectrum unfolding methods in different recovery spectrum as the measurements decreased. The line of blue stars represents the original spectral signal; that is, the Plank spectrum and Triple-peak spectrum. The other lines in different colors and styles represent the recovery signal based on three different methods, namely the Piece-wise B-spline, Gaussian Bump, and Compressive Sensing. m denotes the number of measurements; that is, the number of detection channels.

#### 4.3.1. Spectrum Unfolding Method Contrast: Plank Spectrum

**Figure 5:**
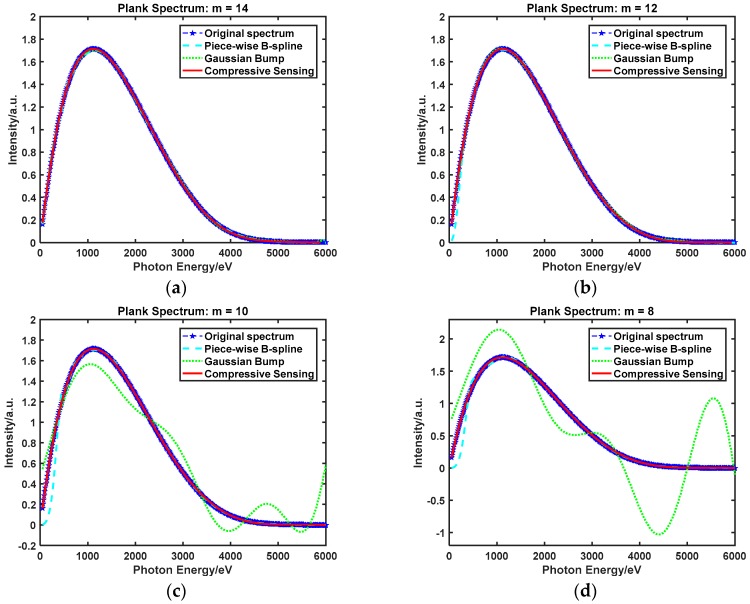
Plank spectrum recovery performance with three different spectrum unfolding methods as the measurements decrease. (**a**) The number of detection channels is 14. (**b**) The number of detection channels is 12. (**c**) The number of detection channels is 10. (**d**) The number of detection channels is 8.

Figure 5 shows the Plank spectrum recovery performance with three spectrum-unfolding methods with respect to a decreasing number of measurements. In Figure 5a, it can be seen that when the number of detection channels is high enough (m=14), good recovery performance can be achieved by all the methods. Figure 5b shows the results of decreasing the number of detection channels to 12. The recovery spectra for two of the methods still keep in line with the original spectrum, however the Piece-wise B-spline is a little under-fitting. Figure 5c shows that when the number of detection channels is reduced to 10, the recovery spectrum with the Gaussian Bump method is under-fitting. A similar result can be seen in Figure 5d, where the number of detection channels is reduced to 8. The different recovery performance of the three recovery spectra is obvious, only the recovery spectrum based on the compressive sensing method stayed in accord with the original spectrum.

To illustrate the problem more evidently, the recovery error was used to show the recovery performance with different spectrum unfolding methods, and RMSE and MAE were calculated by Equations (25) and (26). The smaller the value was, the better the recovery performance that could be achieved. As shown in Table 1 (the smallest value is bold), as the number of detection channels decreases from 14 to 8, the recovery error based on the compressive sensing method is almost unchanged; the RMSE varied from 0.001992 to 0.002013, and the MAE varied from 0.001293 to 0.001315. A significant change took place in both the Piece-wise B-spline and Gaussian Bump methods, for which the RMSE varied from 0.002647 to 0.094138 and 0.007489 to 0.532886, respectively, and the MAE varied from 0.001740 to 0.026802 and 0.006479 to 0.432161, respectively.

The accuracy of the spectrum recovery is reasonable, as the ratio of the error ∆∁ for compressive sensing is 0.31%, which is lower than the 3.5% which was presented to the Plank spectrum recovery in previous work [11]. ∆∁ can be calculated by Equation (27):

(27) $$∆∁=\frac{\sqrt{\frac{1}{n}\sum_{k=1}^{n}{\left(S^{\left(k\right)}-{\hat{S}}^{\left(k\right)}\right)}^{2}}}{\bar{S}}$$

where $\bar{S}$ is the mean of the spectrum, $S^{\left(k\right)}$ is the actual spectrum, ${\hat{S}}^{\left(k\right)}$ is the spectrum unfolding at the kth test point, and n is the number of test points.

**Figure 6:**
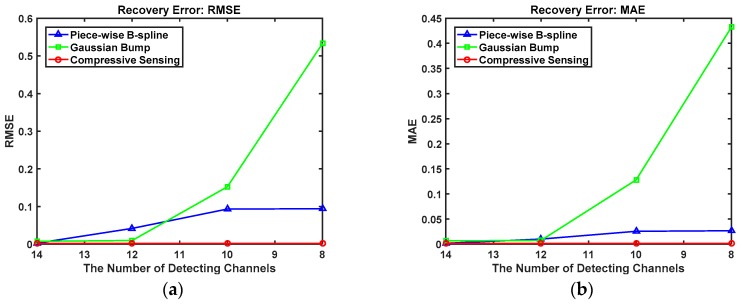
The Plank spectrum recovery error with three different spectrum unfolding methods as the measurements decrease. (**a**) Root mean squared error (RMSE). (**b**) Mean absolute error (MAE).

Figure 6 shows the recovery errors of the three spectrum-unfolding methods with respect to the decreasing number of measurements, which describes the accuracy variation tendency. When the number of measurements was m=8, compressive sensing showed better performance than the other spectrum unfolding methods. Furthermore, it can be seen that the accuracy tendency based on compressive sensing is the most stabilized of these three methods.

#### 4.3.2. Spectrum Unfolding Method Contrast: Triple-Peak Spectrum

**Figure 7:**
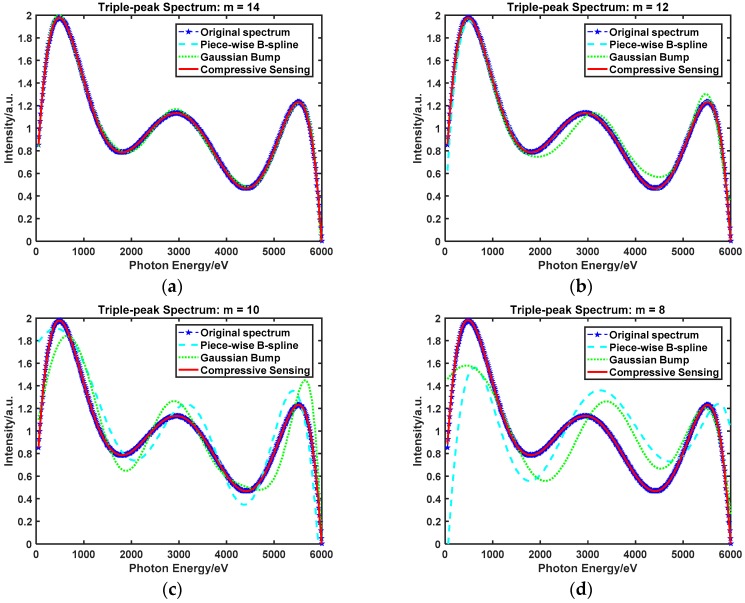
The Triple-peak spectrum recovery performance with three different spectrum unfolding methods as the measurements decrease. (**a**) The number of detection channels is 14. (**b**) The number of detection channels is 12. (**c**) The number of detection channels is 10. (**d**) The number of detection channels is 8.

Figure 7 shows the Triple-peak spectrum recovery performance based on three spectrum-unfolding methods with respect to a decreasing number of measurements. In Figure 7a, the behavior of the three recovery methods is similar. As the number of detection channels decreases from 14 to 8, shown in Figure 7b–d, it can be seen that the recovery performance based on the Piece-wise B-spline and Gaussian Bump methods moves to under-fitting, and only the recovery spectrum based on the compressive sensing method stays in accord with the original spectrum.

Table 2 shows the recovery error of the Triple-peak Spectrum with three spectrum-unfolding methods, with the smallest value in bold. Contrasting in RMSE, the range of the compressive sensing method is [0.001137,0.005037], and it is the lowest of the three methods. The same result is shown when contrasting in MAE, with the lowest range being [0.000116,0.001917].

**Figure 8:**
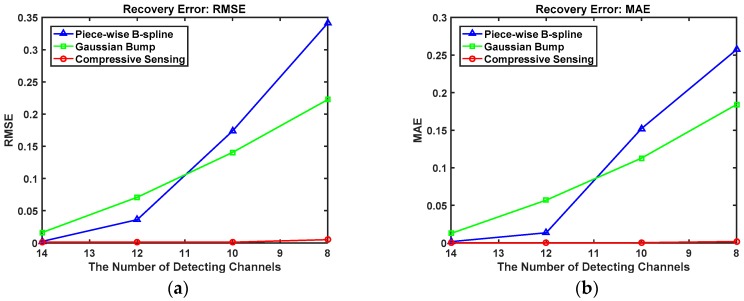
The Triple-peak spectrum recovery error with three different spectrum unfolding methods as the measurements decrease. (**a**) RMSE; (**b**) MAE.

Figure 8 describes the Triple-peak spectrum recovery errors with three different spectrum unfolding methods when the detection channels decrease. As one can see from this figure, the compressive sensing method performs better than the other spectrum unfolding methods.

Throughout the two groups of experiments, the results showed that the spectrum unfolding method based on compressive sensing has the best recovery performance of these methods, and it can still achieve comparable accuracy from only 8 spectrometer detection channels as it has done from 14 detection channels previously; hence, about 42.86% of the space can be saved.

## 5. Conclusions

The laser facility for researching Soft X-ray is a complex system, with limited locations for installing detection channels to obtain measurements, and thus the soft X-ray spectrum can be difficult to recover. In this paper, we proposed a novel recovery method for unfolding Soft X-ray spectrum based on compressive sensing. This method has the following characteristics: (1) Spectrum recovery is formulated as a problem of accurate signal recovery from a few measurements (i.e., compressive sensing); and (2) we use an orthogonal basis function, such as the Legendre polynomials, to sparsely represent the spectral signals, and the corresponding coefficients can be obtained by Lasso regression in the sense of the ℓ1 norm. Since the basis atoms are selected adaptively over the basis dictionary with a large size, the under-fitting problem with limited detection channels is solved, and the spectrometer can achieve higher accuracy spectral information with less measured data.

In order to prove and demonstrate the performance and robustness of the recovery method based on compressive sensing, we conducted self-inspection of the numerical test for recovering two spectra: The Plank spectrum and the Three-peak spectrum. The results show that comparable accuracy can be achieved from only 8 spectrometer detection channels as was previously achieved from 14 detection channels. This means that the presented approach has the capability of recovering a spectrum from limited detection channels data, and it can be used to save more space for other detectors.

## Figures and Tables

**Table 1:** The recovery error of the Plank spectrum with three spectrum-unfolding methods.

M	RMSE			MAE		
	Piece-Wise B-Spline	Gaussian Bump	Compressive Sensing	Piece-Wise B-Spline	Gaussian Bump	Compressive Sensing
14	0.002647	0.007489	**0.001992**	0.001740	0.006479	**0.001293**
12	0.041785	0.009726	**0.002006**	0.010048	0.007416	**0.001305**
10	0.093317	0.152139	**0.002010**	0.025542	0.128225	**0.001311**
8	0.094138	0.532886	**0.002013**	0.026802	0.432161	**0.001315**

**Table 2:** The recovery error of the Triple-peak Spectrum with three spectrum-unfolding methods.

M	RMSE			MAE		
	Piece-Wise B-Spline	Gaussian Bump	Compressive Sensing	Piece-Wise B-Spline	Gaussian Bump	Compressive Sensing
14	0.002298	0.016160	**0.001137**	0.001734	0.012620	**0.000116**
12	0.036192	0.070879	**0.001179**	0.013579	0.056895	**0.000170**
10	0.173786	0.140535	**0.001245**	0.152061	0.112756	**0.000428**
8	0.341228	0.222501	**0.005037**	0.257305	0.184214	**0.001917**
